# Switching and Escalating Therapy in Long-Lasting Multiple Sclerosis: Not Always Necessary

**DOI:** 10.5402/2012/451457

**Published:** 2012-12-22

**Authors:** Ana Teresa Carvalho, Maria José Sá

**Affiliations:** ^1^MS Clinic, Department of Neurology, Centro Hospitalar de São João, 4200-319 Porto, Portugal; ^2^Department of Neurology, Centro Hospitalar de Vila Nova Gaia/Espinho, 4434-502 Vila Nova de Gaia, Portugal; ^3^Faculty of Health Sciences, University Fernando Pessoa, 4249-004 Porto, Portugal

## Abstract

Although therapy switch is common among patients with multiple sclerosis (MS), sometimes the initial prescribed treatment is maintained for a long period with clinical stability, low disability, and nonsignificant side effects. We aim to describe demographic and clinical characteristics of patients treated in our MS clinic with the same disease-modifying drug (DMD) lasting for >12 years. From the cohort of 51 patients followed in our MS clinic with relapse-remitting MS who started an DMD between 1996 and 1999, we found a high percentage (51%) of patients who were efficiently treated with the first DMD. These patients were mainly females, with low annualized relapse rate and Multiple Sclerosis Severity Score (MSSS). Our results may be related to the open and multidisciplinary model of our MS clinic organization. Identifying characteristics associated with therapy persistence may be useful in developing strategies to improve therapy effectiveness.

## 1. Introduction 

Multiple sclerosis is a chronic inflammatory primary demyelinating disease of the central nervous system of unknown etiology; as a result curative therapies have not yet been described [1, 2]. However, in the last two decades, after the discovery and approval of immunomodulators which specifically modify the natural history of the disease, as such denominated disease-modifying drugs (DMDs), most MS patients have been worldwide subjected to this therapy.

Interferon beta (IFN*β*) formulations and glatiramer acetate (GA) are the first-line DMD for MS. IFN*β* is available in five distinct formulations: IFN*β*-1b (Betaferon/Extavia), IFN*β*-1a intramuscular (Avonex), and IFN*β*-1a subcutaneous (Rebif). GA is commercially termed Copaxone. These DMDs were firstly approved to the relapsing-remitting form of MS (RRMS), later for clinically isolated syndromes and IFN*β*-1b for secondary progressive MS (SPMS) [1]. First-line DMDs for MS were introduced in Portugal in 1996, after the licensing of Betaferon for RRMS. The other formulations of IFN for RRMS were introduced later: Avonex in 1997 and Rebif in 1998. Still, Copaxone was introduced in 2001. 

In the last years the therapeutic armamentarium for MS was enlarged, with the approval of drugs with better efficacy yet potential limiting adverse effects, as mitoxantrone (Novantrone), natalizumab (Tysabri), and more recently the oral drug fingolimod (Gilenya). Together, these drugs constitute the second-line treatment for MS and they are usually indicated in more severe non-IFN*β*-responder cases [1, 2]. 

First-line DMDs target multiple neuroimmunological mechanisms implied in the pathogenesis of MS, yet their therapeutic effects are rather modest, mainly traduced by *a* ≈ 30% reduction in the relapse rate and a decrease of magnetic resonance imaging (MRI) T2 lesion burden and gadolinium-enhanced lesions [1, 2]; unfortunately, the benefit effects in disability at long-term remain to be settled. These drugs are usually safe and well tolerated [1]; however, they are not free from side effects.

Failure of the first prescribed DMD was found to occur in 30% of patients usually within 3 years [3]. Therefore, recommendations for escalating therapy were established and successively reviewed by international consensus [4]. Switch to another first- or second-line DMD is common and occurs in a median time of 2.9 years after the initially prescribed DMD; overall, approximately half of MS patients discontinue the use of their DMD within 6 years [5]. Even so, MS neurologists often recognize that, in the clinical setting, some patients maintain the initial treatment for a long period with clinical stability, low disability, and nonsignificant side effects. 

Concerning this issue, which is poorly addressed in the literature, we aim to describe the demographic and clinical characteristics of MSpatients followed in our MS Clinic with long-term(>12 years) maintenance of the first prescribed DMD.

## 2. Patients and Methods

From the database of our MS clinic regarding patients with the definite diagnosis of RRMS (revised McDonald criteria 2005) [6], who started a first-line DMD between 1996 and 1999 (*n* = 51), we identified those who were still receiving the initial therapy in March 2012 (cut-off date).

Inclusion criteria were definite MS diagnosis; RR course at the moment of DMD prescription; age ≥18 years; maintain treatment with first DMD. 

Exclusion criteria were other MS course other than RR when starting DMD; age <18 years; DMD discontinuation; being treated with at least a second DMS. So, switch to another first- or second-line therapy, to a higher dose (in the case of Rebif), or to immunosuppressants, association therapy regimen, were exclusion criteria. 

We proceed to retrospectively review the demographic and clinical protocols of these patients, regarding age, gender, onset syndrome, DMD, annualized relapse rate (ARR) before and after starting treatment, disease duration until March 2012, pregnancy history, Expanded Disability Status Score (EDSS) at treatment start and currently, and the present Multiple Sclerosis Severity Score (MSSS). 

## 3. Results

**Figure 1:**
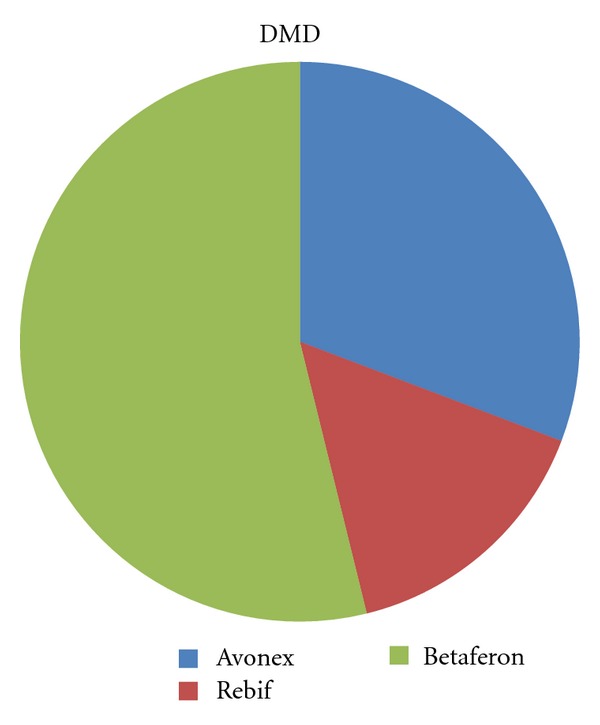
Initial prescribed DMD in patients who never needed to switch/escalate therapy.

**Figure 2:**
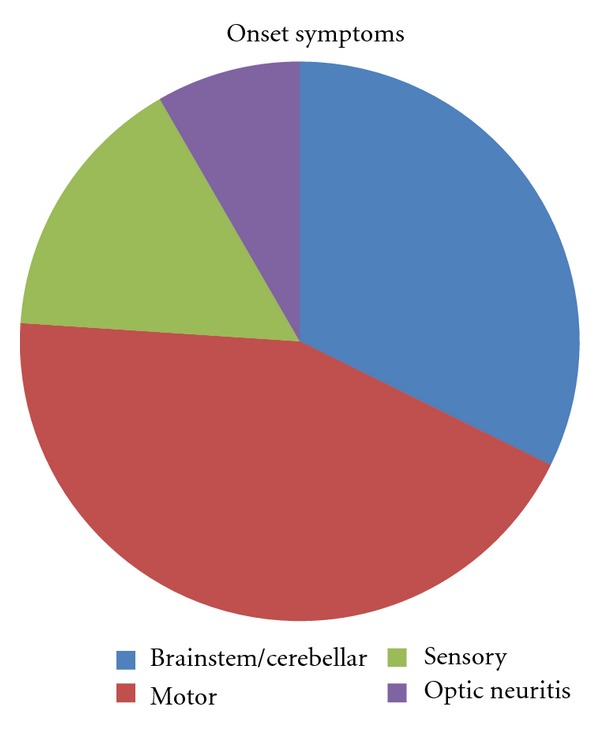
Onset symptoms of patients who never needed to switch/escalate therapy.

From the cohort of RRMS patients who started IFN*β* from 1996 to 1999 (only IFN*β* formulations were available in that time span), we identified 26 cases (51%) treated with the same drug lasting for >12 years (mean = 14 years, range 13–16) (Table 1). Fourteen patients (54%) were treated with Betaferon, 8 (31%) with Avonex and 4 (15%) with Rebif (Figure 1). No serious adverse effects were reported. Concerning gender distribution, 18 patients (69%) were females and 8 males (ratio 2.3 : 1). First symptoms began in the mean age of 31 years (range 16–48), with a mean disease duration of 18.6 years (range 13–32) until March 2012. Regarding clinical presentation, motor symptoms were the most common, occurring in 11 patients (42%); brainstem occurred in 8 (31%), sensory in 4 patients (15%), optic neuritis in 2 (8%), and cerebellar in 1 (4%) (Figure 2). Mean ARR before and after initiating treatment was 0.5 and 0.1, respectively. Median EDSS when starting treatment and currently was 1.5 (range 0–6) and 4 (range 0–6), respectively; present mean MSSS is 2.56 (range 0.10–5.15). Regarding pregnancy, only two women get pregnant during DMD treatment, both under Avonex. Pregnancies developed without complications, deliveries were both normal and newborns were healthy. The remaining women, except one, already had children at the time of starting DMD.

## 4. Discussion 

The management of patients with chronic diseases, as MS, is a major challenge to the physicians. In this sense, the growing appearance of DMD specifically developed for MS with different degrees of efficacy, represents a huge step forward in the management of these patients. However, the success of healthcare relies not only on effective therapies, but also on adherence and persistence. In MS, there is evidence that DMD must be given as early as possible, usually with a first-line drug. Switch to another first-line DMD or escalating therapy to a second-line drug is common, mainly because of lack of efficacy or intolerance. 

Open-label studies found that approximately 30% of MS patients discontinue therapy within 3years due to disease activity[7]; within 6 years, approximately half of the patients discontinue their DMD. In particular, some authors found that 15% and 25% of patients stopped taking the initial DMD after 6 months and 1 year of treatment, respectively; overall, 66% were no longer taking the original DMD at some point of the disease course [5]. Regarding long-lasting treatment with DMD, Evans et al. [5] concluded that patients with a longer disease duration and higher level of disability are at higher risk for discontinuing DMD; age, sex, and the initial DMD were not associated to discontinuation. 

As far as MS is better understood and new drugs with higher efficacy are regularly licensed, the clinicians are concerned with defining better escalating regimens, aiming that patients become “free from disease activity” (no relapses, no progression, and no imagiological activity); consequently, the literature data are mainly focused on those patients who experience a worse treatment response or clinical progression. On the contrary, patients that have an optimal response to the initial prescribed DMD are, apparently, subject of less interest, although this is a crucial issue in order to establish patterns of “good responders.” In fact, data from 260 patients showed that 30% are taking IFN*β*-1b after a median length of exposure of almost 10 years. 

This heterogeneity of treatment response to DMD in MS has beenevaluated by several studies [8–10]. Among DMDs, there seems to be no difference in discontinuation rates [11]. Comparing to nonresponders, responders seem to be older and to have longer disease duration at the time IFN*β* was initiated. Among patients with a RR course, responders have a higher relapse rate during the year prior to IFN*β* therapy. Also, responders seem to have a higher EDSS at initiation of IFN*β* [8]. A probable role of molecular biologic mechanisms contributing to the variable therapeutic response in MS patients has been raised. For example, Cucci et al. demonstrated that proinflammatory cytokine and chemokine mRNA blood level, in MS are related to treatment response [12]. 

Although DMD may reduce the clinical relapse rate, their potential benefits in terms of disability progression may be small. Nevertheless, a favorable treatment response leads the patient and clinician to an expectation of a better prognosis. A multiplicity of variables have been associated with favorable disease course, such as female gender, younger age at onset, sensory symptoms or optic neuritis at onset, and monosymptomatic presentation. In contrast, male gender; onset with motor, sphincter, or cerebellar features; poor recovery from initial or early attacks; higher attack rate in the first 5 years; a progressive course were prognostic variables associated with a poor outcome [13–15]. 

In this study, we found a high percentage of patients who never needed to switch or escalate therapy, some of them treated with the initial prescribed DMD as long as 16 years. Interestingly, our patients had a good outcome despite preponderance of motor and brainstem symptoms at presentation, usually associated with worse prognosis as already stated. Following the same reasoning, we expected a higher prevalence of sensory symptoms or optic neuritis presentation—which in fact occurred only in a minority of our patients. Also, as elsewhere stated, patients who maintain a RR disease course tend to be extremely well and it is only on entering the secondary progressive course of the disease that more rapid disability progression occurs [16]. So, it is possible that our cohort had a good outcome in terms of disability, not only because of the treatment, but also because only patients with RR course (a favorable clinical form) entered the study. We also observed a low EDSS score and a low number of relapses, in agreement with Ramsaransing and De Keyser [14] who found these clinical factors to be predictive of a good outcome at 10 years. 

Another explanationfor ourresultscan berelatedwith a good treatment adherence, defined as “the duration of time from initiation to discontinuation of therapy” [5]; although we have not collected this data in the present cohort, prior results obtained in a larger series of our MS Clinic showed a very low rate of patient's DMD abandon (4.6%), which may be related to the organization model of our MS consultation, as stressed by Castro et al. [17]. This model is based on an open and multidisciplinary care, involving several MS dedicated human resources, namely, neurologists, urologists, psychiatrists, neuropsychologists, social worker, and nurses. There is a personalized training in initial drug injections, complemented with extensive verbal and written information at that time. Also, drugs are provided by the hospital pharmacy in a controlled manner. Further, our MS Clinic provides a permanent (except during night) full access to a MS specialized neurologist. In case of doubts or new symptoms, patients are instructed to access this consultation, instead of emergency room, where misinterpretation of the symptoms is more frequent, particularly when neurologist is not available.

Our small cohort is an obvious limitation of the study; however, it would be rather difficult to find a large number of RRMS patients on a single DMD for such a long period of time.

We are also aware of methodological limitations inherent to observational, retrospectivestudies, such asthe absence of standardizedprotocol. Yet, these kinds of studies provide a demonstration of the real population behavior.

## 5. Conclusion

We found a high percentage of patients who never needed to switch or escalate therapy, who were mainly females, with low ARR, EDSS, and MSSS after long disease duration. Identifying characteristicsassociated with therapy persistencemay beuseful in developingstrategies to improve therapy effectiveness.

## Figures and Tables

**Table 1:** Characteristics of multiple sclerosis patients efficiently treated with the first prescribed immunomodulator for >12 years.

	Patient	Gender	Age of onset (years)	Onset symptoms	DMD	Treatment duration (years)	Disease duration (years)	MSSS
	1	F	34	Motor	Avonex	14	15	2.82
	2	F	39	Motor	Betaferon	16	17	3.65
	3	F	19	Brainstem	Avonex	14	33	0.01
	4	M	24	Brainstem	Betaferon	14	22	3.69
	5	F	45	Brainstem	Avonex	15	15	0.99
	6	F	22	Brainstem	Betaferon	14	16	1.42
	7	F	20	Sensory	Avonex	13	14	0.49
	8	F	45	Sensory	Betaferon	13	15	414
	9	F	40	Cerebellar	Avonex	13	18	3.37
	10	M	16	Brainstem	Betaferon	14	27	2.56
	11	M	21	Sensory	Betaferon	13	18	3.37
	12	F	26	Motor	Betaferon	14	28	1.16
	13	F	41	Motor	Betaferon	13	15	4.68
	14	F	?	Sensory	Betaferon	14	?	?
	15	M	40	Motor	Betaferon	16	18	0.26
	16	M	32	Motor	Betaferon	14	16	4.81
	17	F	35	Motor	Betaferon	15	20	5.15
	18	M	38	Optic neuritis	Betaferon	13	13	0.13
	19	F	48	Optic neuritis	Avonex	13	14	0.49
	20	F	39	Motor	Avonex	13	15	0.45
	21	F	23	Motor	Rebif 22	13	24	5.03
	22	F	40	Brainstem	Rebif 22	13	14	4.26
	23	M	27	Brainstem	Rebif 22	13	15	0.10
	24	M	17	Motor	Rebif 44	13	27	2.56
	25	F	18	Motor	Betaferon	14	32	2.23
	26	F	29	Brainstem	Avonex	15	18	3.89

Mean value (if applicable)	—	—	31	—	—	14	18.6	2.56

EDSS: expanded disability status scale; F: female; M: male; MSSS: multiple sclerosis severity score; ?: missing data.
